# Supplementary material for: Network analysis of cross-income-level collaboration on multiple myeloma in sub-Saharan Africa
Source: Npj Health Syst. 2025 Jan 7;2:1. doi: 10.1038/s44401-024-00003-2 (PMC13354229; doi:10.1038/s44401-024-00003-2)
Supplement: Supplementary file 1 — Supplementary Information [file 44401_2024_3_MOESM1_ESM.pdf]

Supplementary File

Kaiyi Yang, Sam Benkwitz-Bedford, Jean-Baptiste Cazier and Fabian Spill

1 Publication landscape

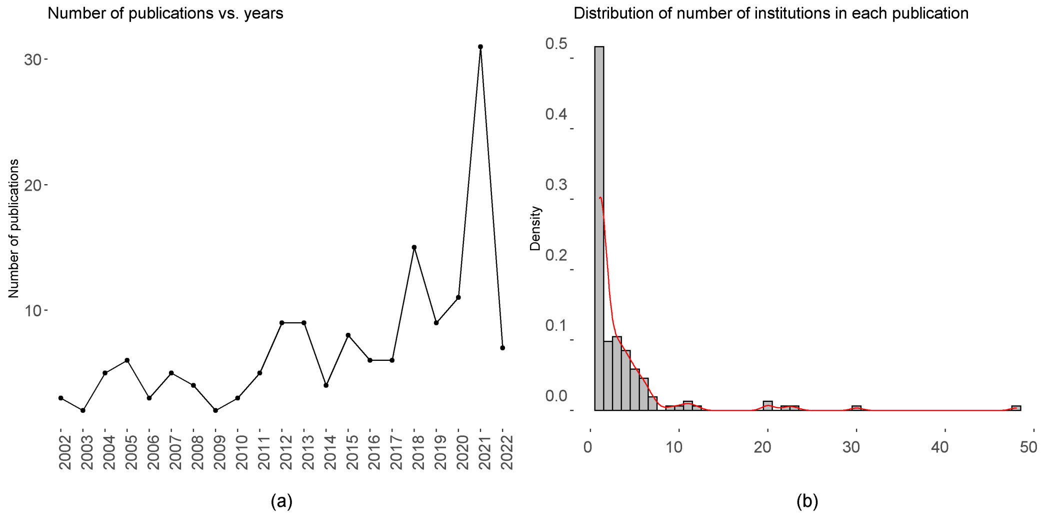

Supplementary Figure 1: **Publication statistics.** (a) Line graph of the number of publications by year (as of June 2022) and (b) Histogram of the number of institutions in each publication (2002-2022).

2 Country-level collaboration network

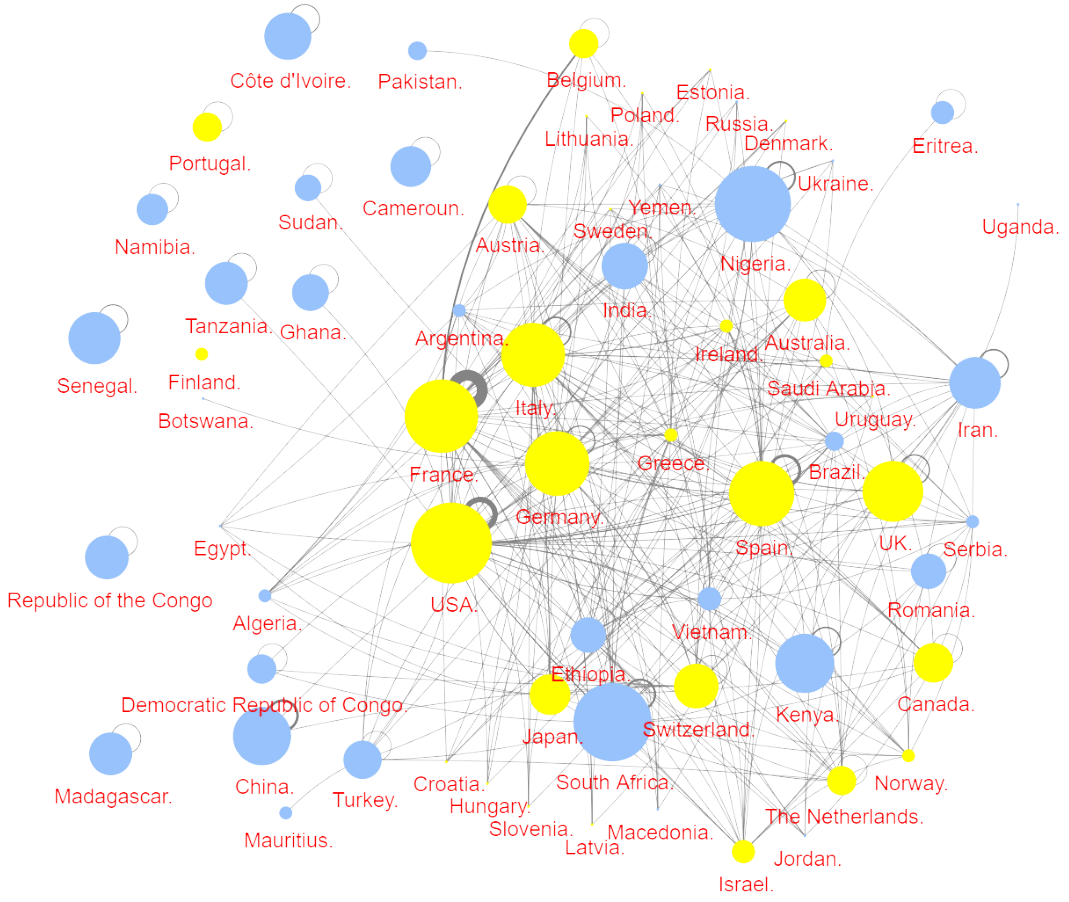

Supplementary Figure 2: **Country level collaboration network.** LMICs in blue, HICs in yellow. The size of the nodes is proportional to the number of authors in that country who have publications in the field, and the thickness of the edges is proportional to the strength of the collaboration.

3 Institution level

3.1 Institution-level metrics

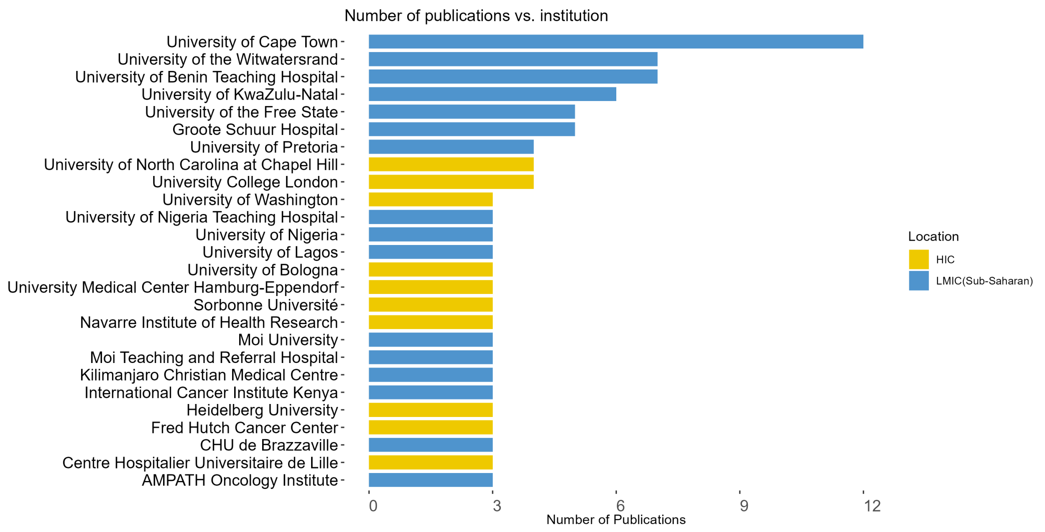

Supplementary Figure 3: **Bar charts of the number of publications on MM in SSA by institutions (top 26) between 2002 and 2022.**

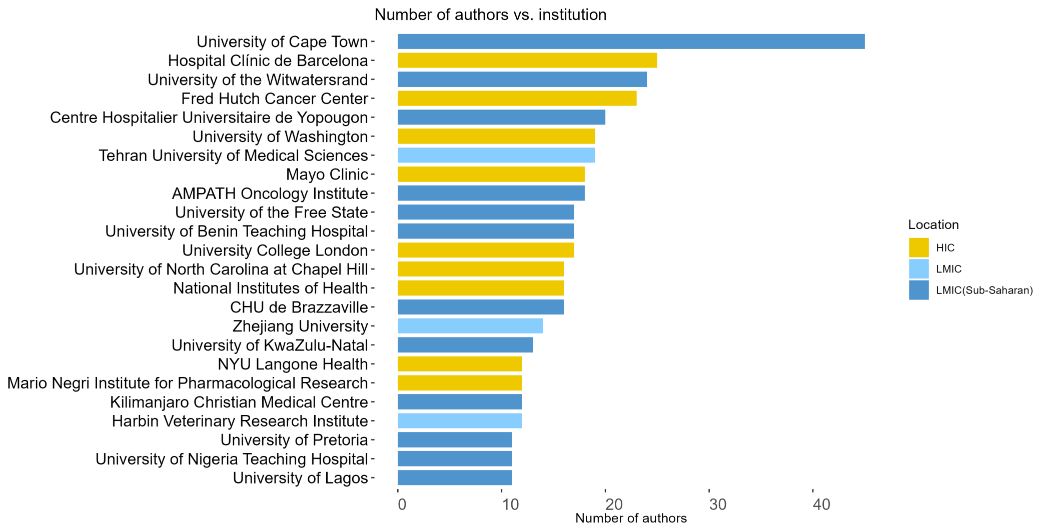

Supplementary Figure 4: **Bar charts of the number of authors by institutions (top 24).**

3.2 Complete institution-level collaboration network

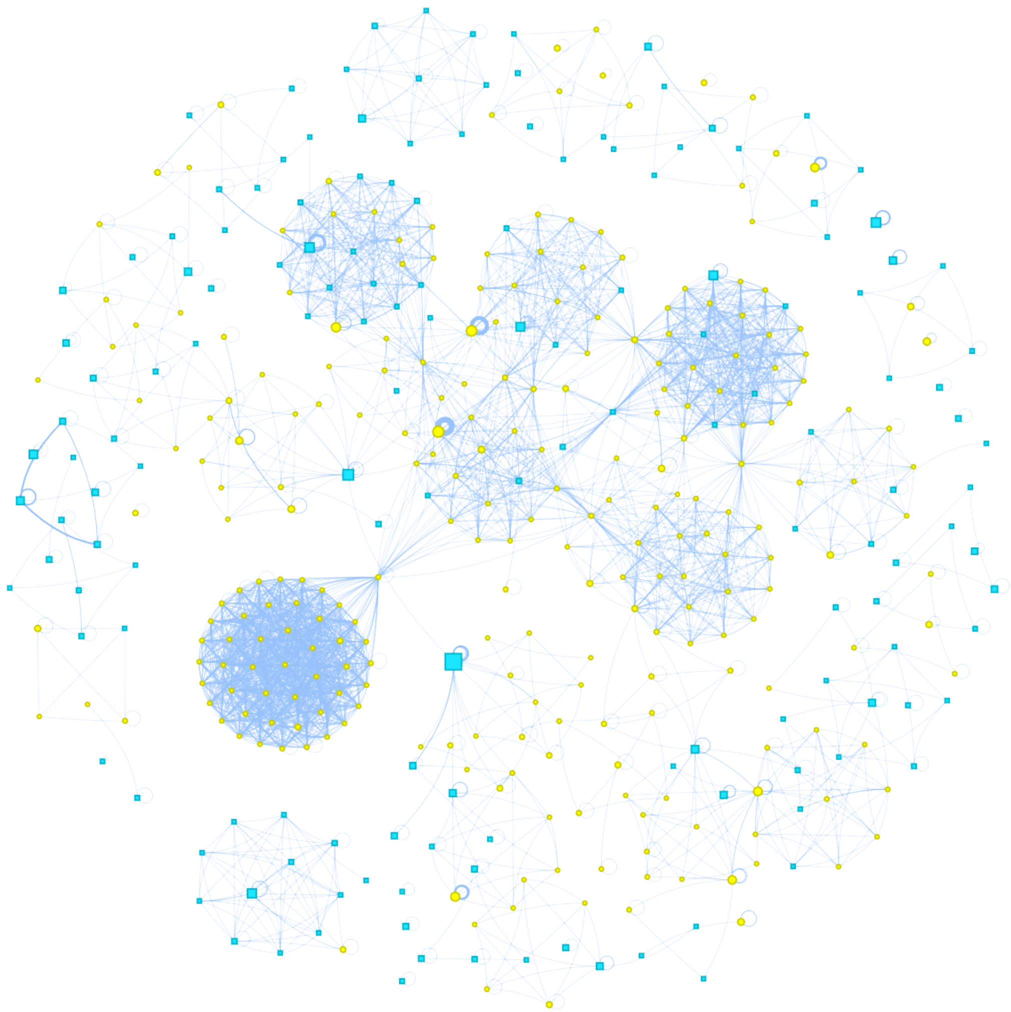

Supplementary Figure 5: **The full collaboration network at the institution level.** Institutions in HICs are in yellow, and institutions in LMICs are in blue. Node size is proportional to the number of authors. The thickness of the edges is proportional to the strength of collaboration.

4 Subnetwork –Clinical trial and observational study publications

We selected publications explicitly tagged as 'clinical trial' and 'observational study' on PubMed, seven in all. The sub-network was constructed similarly, As we can see from Supplementary Figure 6, institutions from HICs were overwhelmingly dominant in this sub-network, a trend that is even more pronounced than the one shown in the full network graph. In light of this phenomenon, we scrutinised relevant publications, such as the clinical trial publication with PMID 26089396, which lists authors with affiliations from various hospitals in Spain, and finally, a large collaborative group (the appendix of this publication lists some collaborators from local hospitals in SSA), which suggests that local institutions were indeed involved in the study but their participation is limited. Among the few institutions from , only a few institutions from South Africa are local, which highlights the special role of South Africa.

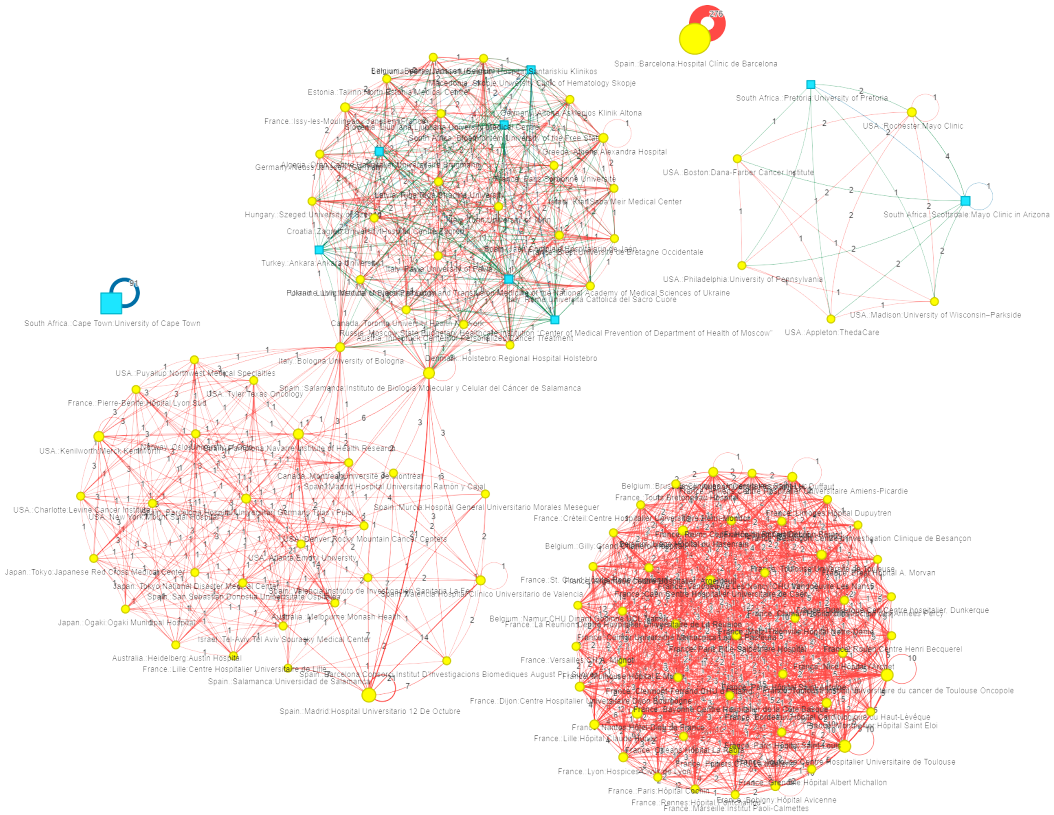

Supplementary Figure 6: **Clinical trial and observational study publications – institutional collaboration network graph.** Vertex colours: Institutions from LMICs in blue, Institutions from HICs in yellow; Edge colours: LMLM (Intra-group collaboration among institutions in LMICs) in blue, LMHC (CILC) in green, HCHC (Intra-group collaboration among institutions in HICs) in red. The number of contributions from affiliated authors determines the node size and the strength of collaboration determines the thickness of the edge

5 Betweenness centrality and eigenvector centrality top institutions identified

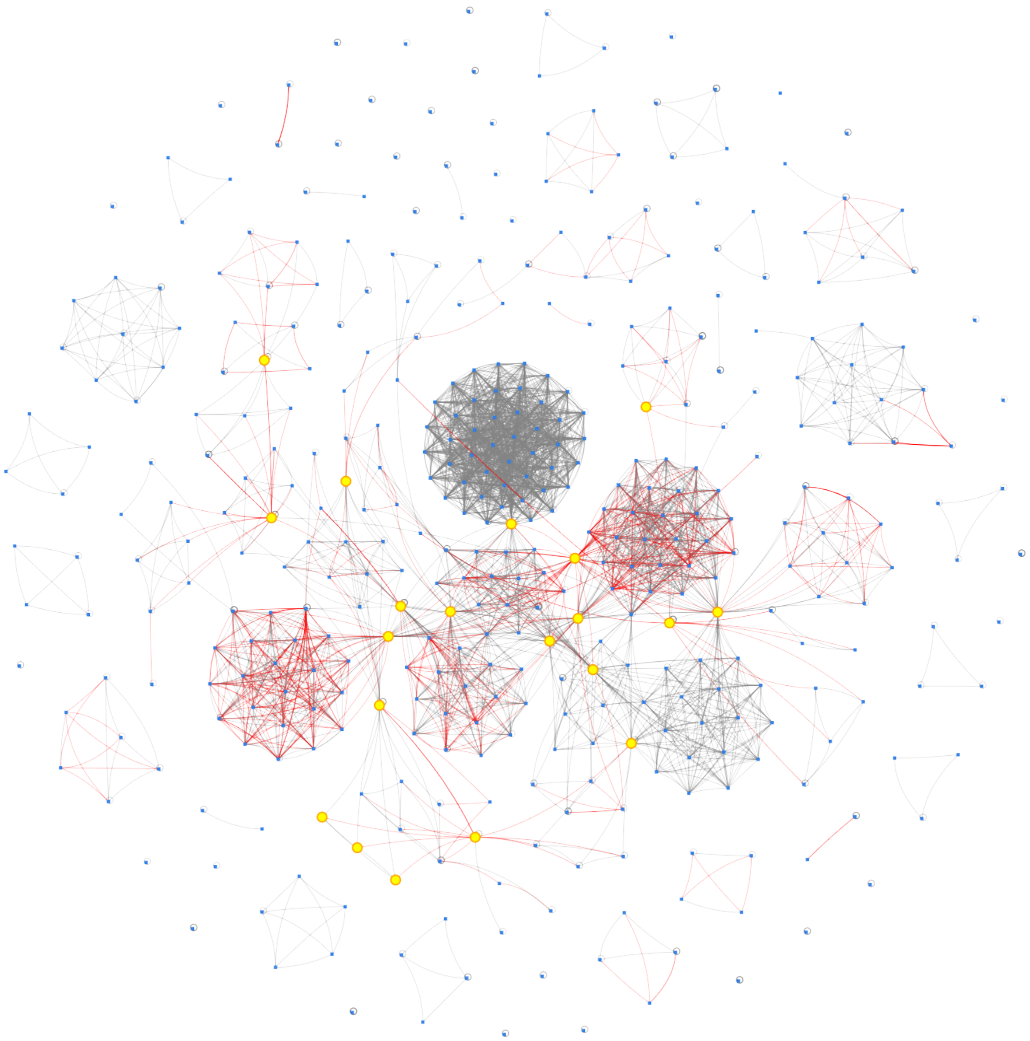

Supplementary Figure 7: **Institutional network graph for betweenness centrality.** The top 20 institutions identified by betweenness centrality are highlighted. The edges representing the cross-group collaboration are in red.

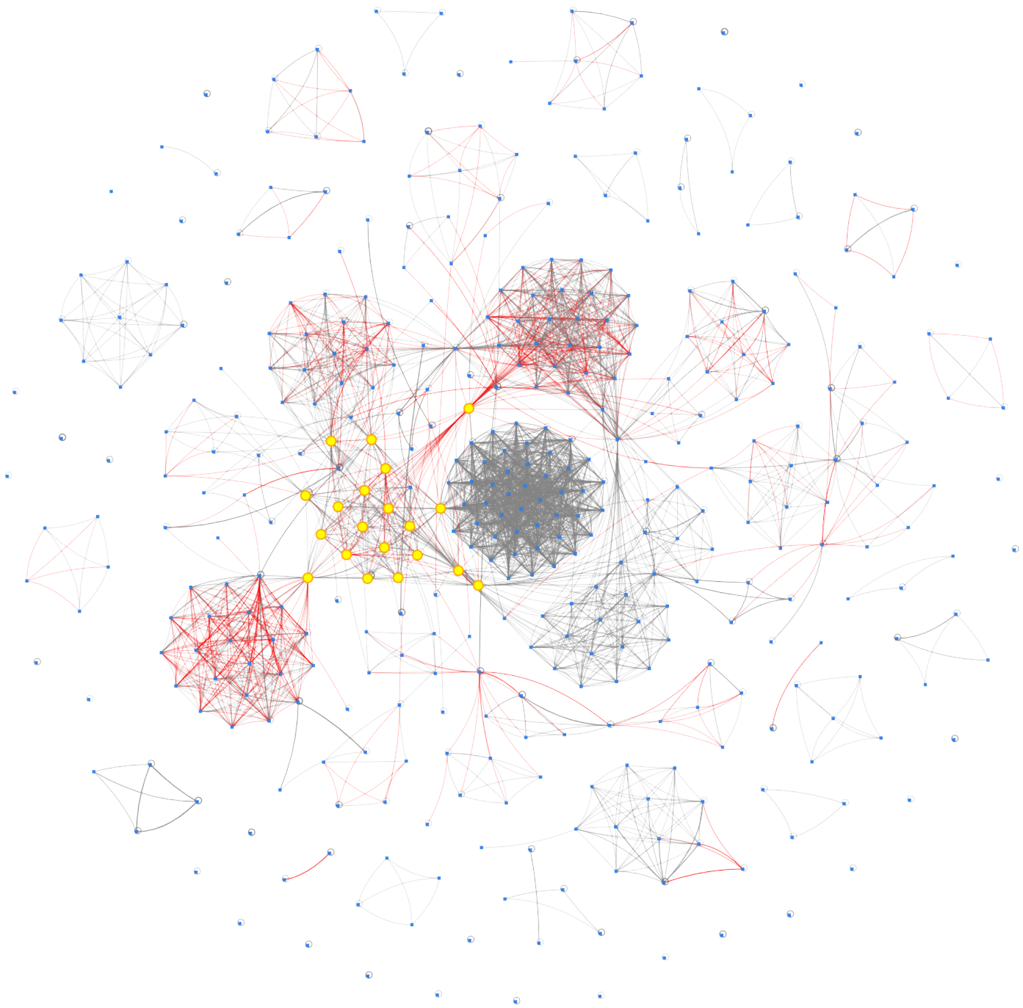

Supplementary Figure 8: **Institutional network graph for eigenvector centrality.** The top 20 institutions identified by eigenvector centrality are highlighted. The edges representing the cross-group collaboration are in red.

6 Adjusted degree centrality ranking of institutions

The complete results of the ranking for different values of α are shown in Supplementary Table 1.

Supplementary Table 1: Ranking of institutions when different tuning parameter values are used. (A smaller α indicates centrality is mainly determined by extensiveness, while a larger α indicates centrality is mainly determined by strength.)

R	α=0.2	α=0.5	α=0.8
1	University of the Witwatersrand	University of Washington	University of Washington
2	University of the Free State	University of the Witwatersrand	Fred Hutch Cancer Center
3	Ankara University	Ankara University	University of Cape Town
4	University of Benin Teaching Hospital	University of Cape Town	University of the Witwatersrand
5	University of Washington	Netcare Pretoria East Hospital	Netcare Pretoria East Hospital
6	University of KwaZulu-Natal	Fred Hutch Cancer Center	Hospital Clínic de Barcelona
7	Netcare Pretoria East Hospital	University of the Free State	Tehran University of Medical Sciences
8	University of Cape Town	University of KwaZulu-Natal	Indiana University School of Medicine
9	Fred Hutch Cancer Center	Tehran University of Medical Sciences	University of KwaZulu-Natal
10	Sorbonne Université	University of Benin Teaching Hospital	Ankara University
11	Tehran University of Medical Sciences	Hospital Clínic de Barcelona	AMPATH Oncology Institute
12	University of Bologna	Sorbonne Université	University College London
13	Hospital Clínic de Barcelona	University College London	University of the Free State
14	Institut Paoli-Calmettes	AMPATH Oncology Institute	Centre Hospitalier Universitaire de Toulouse
15	University College London	Centre Hospitalier Universitaire de Toulouse	Institut universitaire du cancer de Toulouse Oncopole

Rankings of the top 15 local institutions located in SSA are in Supplementary Table 2.

Supplementary Table 2: Main metrics of the top 15 SSA institutions at $\alpha = 0.5$

Institution	Country	No.A	No.P	D_LH	D_O	S_LH	S_O	Centrality
University of the Witwatersrand	South Africa.	24	7	16	3	49	64	617.0
University of Cape Town	South Africa.	45	12	10	5	31	171	505.1
Netcare Pretoria East Hospital	South Africa.	4	2	19	4	50	6	476.1
University of the Free State	South Africa.	17	5	24	7	26	31	464.7
University of KwaZulu-Natal	South Africa.	13	6	12	3	43	19	450.8
University of Benin Teaching Hospital	Nigeria.	17	7	16	5	20	20	344.7
AMPATH Oncology Institute	Kenya.	18	3	1	12	33	169	221.8
North-West University	South Africa.	3	2	5	2	17	1	94.8
University of Nigeria	Nigeria.	10	3	5	3	12	20	92.8
Haramaya University	Ethiopia.	2	1	10	14	42	29	86.8
Moi University	Kenya.	5	3	1	12	12	49	84.8
University of Pretoria	South Africa.	11	4	5	4	6	18	80.1
Moi Teaching and Referral Hospital	Kenya.	4	3	1	12	6	50	72.4
Echolab Radiology And Laboratory Services, Benin City	Nigeria.	1	1	9	0	11	0	68.6
University of Lagos	Nigeria.	11	3	2	5	6	33	66.5

7 Original degree centrality results

Using the degree centrality function:

$$C_D^{\omega\alpha}(v_i) = \deg(v_i)^{1-\alpha} \cdot S(v_i)^{\alpha}, \alpha \in [0,1] \tag{1}$$

The results of the sorting for different values of α are shown in Supplementary Table 3.

Supplementary Table 3: Ranking of institutions for different values of tuning parameter α

R	α=0.2	α=0.5	α=0.8
1	Institut Paoli-Calmettes.	CHU de Toulouse.	CHU de Toulouse.
2	CHU de Toulouse.	IUCT-O.	IUCT-O.
3	IUCT-O.	CHU Hôtel Dieu.	University of Washington.
4	CHU Hôtel Dieu.	University of Washington.	Hospital Clinic Barcelona.
5	CHRU-Hôpital Claude Huriez.	Institut Paoli-Calmettes.	Tehran University of MeSc.
6	CHU Amiens sud.	Hospital Clinic Barcelona.	CHU Hôtel Dieu.
7	CHU Côte de Nacre.	Tehran University of MeSc.	Zhejiang University.
8	CHU Dijon.	CHRU-Hôpital Claude Huriez.	F.H. Cancer Research Center.
9	CHU Haut Lévêque.	CHU Amiens sud.	AMPATH Oncology Institute.
10	CHU La Mileterie.	CHU Côte de Nacre.	CHRU-Hôpital Claude Huriez.
11	Clinics universities Saint-Luc.	CHU Dijon.	CHU Amiens sud.
12	Hematology Hôpital Avicenne.	CHU Haut Lévêque.	CHU Côte de Nacre.
13	Hospices Civils de Lyon.	CHU La Mileterie.	CHU Dijon.
14	Université de Toulouse.	Clinics universities Saint-Luc.	CHU Haut Lévêque.
15	Ankara University.	Hematology Hôpital Avicenne.	CHU La Mileterie.

From Supplementary Table 4, Supplementary Table 5 and Supplementary Table 6, We note that the smaller tuning parameter α allows the degree centrality to be determined mainly by the number of degrees, while institutions with larger degrees are those in HICs, and as the value of the tuning parameter α increases, some institutions with higher numbers of authors move up in the rankings. In addition to this, we find that for the three different tuning parameters, there are institutions ranked in the top 15 in each case, such as CHU de Toulouse, IUCT-O (short for Institut Universitaire du Cancer de Toulouse-Oncopole), CHU Hôtel Dieu, all of which are French institutions.

These institutions in Supplementary Figure 9 form a non-open cluster, i.e. a large number of French institutions have a lot of mutual cooperation with a few Belgian institutions (which explains their high degrees), but they do not cooperate with institutions from other countries, let alone those from LMICs, as can also be seen from the LMHC column in the Supplementary Table 4. The ranking

Supplementary Table 4: Main metrics of the top 15 institutions at α=0.2

Institution	Country	Type	No.au	Deg	HCHC	LMHC	LMLM	Centrality
Institut Paoli-Calmettes.	France.	HC	3	70	67	3	0	77.44084
CHU de Toulouse.	France.	HC	5	49	49	0	0	71.53746
IUCT-O.	France.	HC	5	49	49	0	0	71.53746
CHU Hôtel Dieu.	France.	HC	3	49	49	0	0	64.7873
CHRU-Hôpital Claude Huriez.	France.	HC	2	49	49	0	0	59.83113
CHU Amiens sud.	France.	HC	2	49	49	0	0	59.83113
CHU Côte de Nacre.	France.	HC	2	49	49	0	0	59.83113
CHU Dijon.	France.	HC	2	49	49	0	0	59.83113
CHU Haut Lévêque.	France.	HC	2	49	49	0	0	59.83113
CHU La Mileterie.	France.	HC	2	49	49	0	0	59.83113
Clinic university Saint-Luc.	Belgium.	HC	2	49	49	0	0	59.83113
Hematology Hôpital Avicenne.	France.	HC	2	49	49	0	0	59.83113
Hospices Civils de Lyon.	France.	HC	2	49	49	0	0	59.83113
Université de Toulouse.	France.	HC	2	49	49	0	0	59.83113
Ankara University.	Turkey.	LMIC	2	50	0	42	8	51.14623

Supplementary Table 5: Main metrics of the top 15 institutions at α=0.5

Institution	Country	Type	No.au	Deg	HCHC	LMHC	LMLM	Centrality
CHU de Toulouse.	France.	HC	5	49	49	0	0	126.1943
IUCT-O.	France.	HC	5	49	49	0	0	126.1943
CHU Hôtel Dieu.	France.	HC	3	49	49	0	0	98.49873
University of Washington.	USA.	HC	19	27	13	14	0	96.79359
Institut Paoli-Calmettes.	France.	HC	3	70	67	3	0	90.11104
Hospital Clinic Barcelona.	Spain.	HC	25	23	20	3	0	83.20457
Tehran University of MeSc.	Iran.	LMIC	19	26	0	10	16	82.37718
CHRU-Hôpital Claude Huriez.	France.	HC	2	49	49	0	0	80.72794
CHU Amiens sud.	France.	HC	2	49	49	0	0	80.72794
CHU Côte de Nacre.	France.	HC	2	49	49	0	0	80.72794
CHU Dijon.	France.	HC	2	49	49	0	0	80.72794
CHU Haut Lévêque.	France.	HC	2	49	49	0	0	80.72794
CHU La Mileterie.	France.	HC	2	49	49	0	0	80.72794
Clinic university Saint-Luc.	Belgium.	HC	2	49	49	0	0	80.72794
Hematology Hôpital Avicenne.	France.	HC	2	49	49	0	0	80.72794

of the institutions in that cluster above is inflated, considering that cooperation between institutions in HICs and those in LMICs is rare but valuable and that such institutions are more useful for potential future cross-income-level cooperation. So the original degree centrality is not a good choice here.

Supplementary Table 6: Main metrics of the top 15 institutions at α=0.8

Institution	Country	Type	No.au	Deg	HCHC	LMHC	LMLM	Centrality
CHU de Toulouse.	France.	HC	5	49	49	0	0	222.6106
IUCT-O.	France.	HC	5	49	49	0	0	222.6106
University of Washington.	USA.	HC	19	27	13	14	0	208.2267
Hospital Clinic Barcelona.	Spain.	HC	25	23	20	3	0	179.9694
Tehran University of MeSc.	Iran.	LMIC	19	26	0	10	16	164.5535
CHU Hôtel Dieu.	France.	HC	3	49	49	0	0	149.7516
Zhejiang University.	China.	LMIC	14	5	0	0	5	132.6317
F.H. Cancer Research Center.	USA.	HC	20	5	4	1	0	121.5864
AMPATH Oncology Institute.	Kenya.	LMIC	18	13	0	1	12	116.6997
CHRU-Hôpital Claude Huriez.	France.	HC	2	49	49	0	0	108.9232
CHU Amiens sud.	France.	HC	2	49	49	0	0	108.9232
CHU Côte de Nacre.	France.	HC	2	49	49	0	0	108.9232
CHU Dijon.	France.	HC	2	49	49	0	0	108.9232
CHU Haut Lévêque.	France.	HC	2	49	49	0	0	108.9232
CHU La Mileterie.	France.	HC	2	49	49	0	0	108.9232

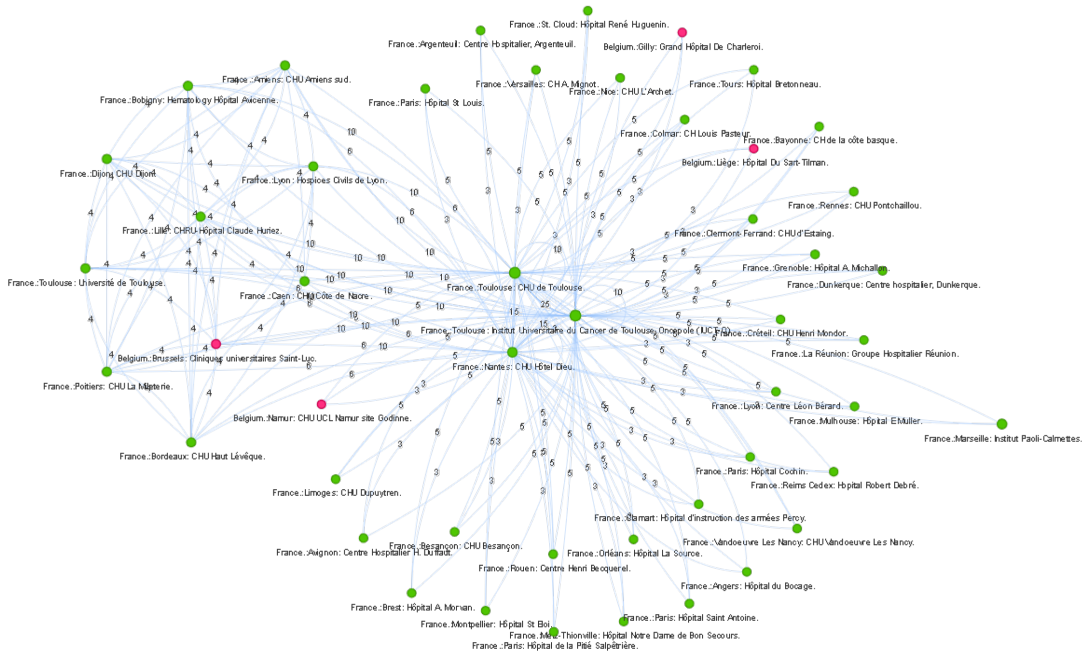

Supplementary Figure 9: **Sub-graph of the French cluster.** Edges with weights less than or equal to 2 have been omitted from the network to focus on highly connected institutions.
